# Supplemental Feeding for Ecotourism Reverses Diel Activity and Alters Movement Patterns and Spatial Distribution of the Southern Stingray, *Dasyatis americana*

**DOI:** 10.1371/journal.pone.0059235

**Published:** 2013-03-18

**Authors:** Mark J. Corcoran, Bradley M. Wetherbee, Mahmood S. Shivji, Matthew D. Potenski, Demian D. Chapman, Guy M. Harvey

**Affiliations:** 1 The Guy Harvey Research Institute, Nova Southeastern University, Dania Beach, Florida, United States of America; 2 Department of Biological Sciences, University of Rhode Island, Kingston, Rhode Island, United States of America; Aristotle University of Thessaloniki, Greece

## Abstract

Southern stingrays, *Dasyatis americana*, have been provided supplemental food in ecotourism operations at Stingray City Sandbar (SCS), Grand Cayman since 1986, with this site becoming one of the world’s most famous and heavily visited marine wildlife interaction venues. Given expansion of marine wildlife interactive tourism worldwide, there are questions about the effects of such activities on the focal species and their ecosystems. We used a combination of acoustic telemetry and tag-recapture efforts to test the hypothesis that human-sourced supplemental feeding has altered stingray activity patterns and habitat use at SCS relative to wild animals at control sites. Secondarily, we also qualitatively estimated the population size of stingrays supporting this major ecotourism venue. Tag-recapture data indicated that a population of at least 164 stingrays, over 80% female, utilized the small area at SCS for prolonged periods of time. Examination of comparative movements of mature female stingrays at SCS and control sites revealed strong differences between the two groups: The fed animals demonstrated a notable inversion of diel activity, being constantly active during the day with little movement at night compared to the nocturnally active wild stingrays; The fed stingrays utilized significantly (*p*<0.05) smaller 24 hour activity spaces compared to wild conspecifics, staying in close proximity to the ecotourism site; Fed stingrays showed a high degree of overlap in their core activity spaces compared to wild stingrays which were largely solitary in the spaces utilized (72% vs. 3% overlap respectively). Supplemental feeding has strikingly altered movement behavior and spatial distribution of the stingrays, and generated an atypically high density of animals at SCS which could have downstream fitness costs for individuals and potentially broader ecosystem effects. These findings should help environmental managers plan mitigating measures for existing operations, and develop precautionary policies regarding proposed feeding sites.

## Introduction

Ecotourism involving wildlife observation in general is a large and rapidly growing industry, generating over US$165 billion annually worldwide [1], [2], [3], [4]. Tourism centered on marine wildlife specifically is also experiencing burgeoning growth given substantial associated economic benefits, and is involving an increasing diversity of species [5]. Because wildlife observations can be unpredictable, ecotourism operators often use food as an attractant for animals to increase encounter rates [6], and in some cases to enhance the tourist experience by allowing them to feed the animals directly [7], [8], [9], [10], [11]. Feeding of terrestrial wildlife is being increasingly regulated, however, because of the many instances where such activities are documented to alter the behavior, population size, reproduction, migration and/or health of the animals being fed, and in some instances even jeopardize human safety (see [6] for a review). In contrast, with the exception of cases involving marine mammals which are often protected under national policy guidelines, there are comparatively few regulations governing the feeding of other marine wildlife, even though the effects of this supplemental feeding on the animals are seldom known.

In the few cases where the effects of supplemental feeding on marine teleost fishes has been examined, changes in behavior, abundance and population structure of individual species, and spatiotemporal characteristics of fish assemblages on a scale of hundreds of meters and many months have been documented [10], [12], [13], [14]. The handful of studies examining the effects of ecotourism feeding on different behavioral aspects of elasmobranchs have suggested variable outcomes, with minor to no apparent effects on some species (white sharks, [15]; Caribbean reefs sharks, [16]), but detectable changes in the behavior of others (pink whipray, [11]; sicklefin lemon sharks, [17]; whitetip reef sharks, [18]).

The supplemental feeding of marine stingrays (subclass Elasmobranchii) is now a common ecotourist attraction in several parts of the world [9], [11], [19]. Due to its abundance and opportunistic feeding habits, the southern stingray, *Dasyatis americana*, is rapidly becoming one of the mainstay tourist attractions at feeding sites in the Caribbean. The longest established and possibly largest such site in the Caribbean is the Stingray City Sandbar location in the Cayman Islands. Stingrays are thought to have been fed in this vicinity dating back to the 1930’s when fishermen would clean their catch near the location. Tourist operations were moved to a nearby shallow sandbar (hereafter SCS) to allow people to stand in the water rather than having to snorkel or dive, and intentional supplemental feeding of *D. americana* has been ongoing at this site since 1986 [7]. The SCS location has gained worldwide recognition and has been referred to as the most popular and successful dive site in the world, receiving over a million tourist visitors per year who feed and otherwise physically interact with (e.g., touch) the stingrays (Ebanks-Petrie, Cayman Islands Department of Environment, personal communication). With the advent of larger scale, organized ecotourism activities at SCS, the stingrays are now almost exclusively fed a non-natural diet of packaged California squid, *Loligo opalescens*, provided by tour operators. The almost daily feeding has also resulted in a large number of stingrays being conditioned to approach (even sometimes “mob”) humans handing out the food, and created a regime of concentrated food availability in a small area during the daytime to facilitate tourist encounters.

The long-term ecotourism, including supplementary stingray feeding, at the SCS site has raised questions and concerns about the effects of these activities on the stingray population [18], [20]. Despite these concerns, the enormous economic benefits of stingray ecotourism in the Cayman Islands has prompted development of several other such programs throughout the Caribbean, and calls for establishing even more such locations in the Cayman Islands and elsewhere [21]. Because marine wildlife interactive experiences (including animal feeding) are proliferating worldwide but remain highly controversial [22], it is valuable to understand the effects these activities may be having on the focal species as well as other organisms with which they normally interact ecologically [10], [15], [23].

Previous studies at SCS have found that the fed stingrays display markers of suboptimal physiological condition and different fatty acid profiles compared to their wild conspecifics [19], [24], [25]. Here, we examine the effects of this regular supplemental feeding on the movements, spatial habitat use and some aspects of the population dynamics of *Dasyatis americana* at the SCS site in Grand Cayman. Because the search for food is likely to be a major element influencing the movement patterns and activity space of most mobile animals [2], [26], [27], we tested the hypothesis that the almost daily feeding by humans has altered activity patterns and habitat use of stingrays at SCS relative to their conspecifics at control (non-supplemental feeding) sites. We document that supplementary feeding by humans has resulted in strong modifications of movement patterns and space utilization by the stingrays.

## Methods

### Ethics Statement

This study was conducted with approval from the Cayman Islands Government Department of Environment and Nova Southeastern University. At the time this field work (animal handling) was performed in 2002–2003, Nova Southeastern University did not have an Animal Welfare Committee and an institutional permit was not required. Rather, all animal handling procedures were conducted using guidelines established by the American Fisheries Society and American Society of Ichthyology and Herpetology, and all efforts were made to minimize animal stress and suffering.

### Study Animals and Sites

**Figure 1:**
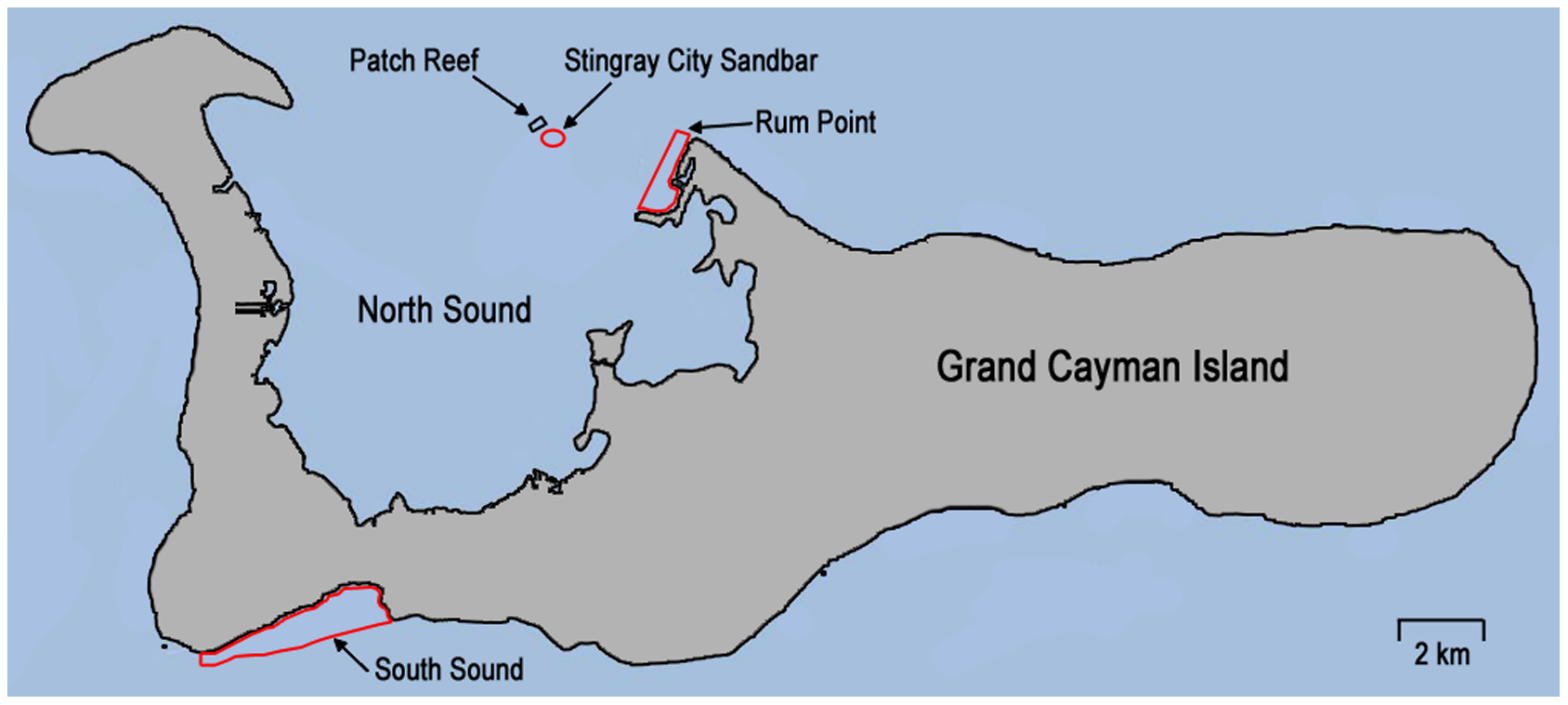
Map of Grand Cayman Island showing study sites. Indicated are the locations of the supplemental feeding site Stingray City Sandbar (SCS), the two control sites South Sound and Rum Point, and a patch reef where female stingrays aggregated at night.

Two groups of stingrays were tracked using manual and automated acoustic telemetry: 1) Stingrays (hereafter “fed” stingrays) captured at SCS, a naturally occurring sandbar located in the North Sound lagoon of Grand Cayman, where human feeding of stingrays occurs on nearly a daily basis; and 2) Stingrays not fed by humans (hereafter “wild” stingrays) at two control sites, the South Sound and Rum Point (Fig. 1).

SCS has a surface area of approximately 0.0078 km^2^ and water depth as shallow as 0.5 m. This site is bordered to the south by a vast *Thalassia testudinum* dominated seagrass plain and to the north by relatively deeper, fringing, patch reefs. The two control sites have a similar overall habitat. The main control site, the South Sound, covers an area of 3 km^2^ forming a semi-enclosed lagoon enclosed by a fringing reef, which opens at the western edge and through an artificial channel in the center of the reef (Fig. 1). Water depth varies from 0.2–3.0 m within the lagoon. A sand flat covers a large portion of the southeastern lagoon, and dense seagrass plains of *T. testudinum* occur in much of the remaining sections of the lagoon. The entire South Sound is designated a “marine replenishment zone” or marine protected area (MPA) by the CIDOE, and was chosen as the primary control site for our study because of its environmental similarity to the North Sound, and the presence of an accessible population of wild, mature female southern stingrays.

The secondary control site, Rum Point, is located at the northeast tip of the North Sound and is enclosed in a MPA. This area also contains mainly sand flat and seagrass plain communities, and covers approximately 0.64 km^2^ with water depths varying from 0.2–2 m. Rum Point is also a nursery for southern stingrays (M. Corcoran, personal observations).

The fed stingrays were captured at SCS with hand-held dip nets and transferred into a seawater-filled canvas pool inside a boat for processing. Wild stingrays were captured at control sites by visually locating them from a boat, encircling them in a hand-drawn seine net and transferring them to the boat with a dip net. Capture location of each wild animal caught was recorded using a handheld GPS. For each fed or wild stingray processed, sex was recorded and disc width (±1 cm) measured using a tape measure across the broadest extent of the pectoral fins. A passive integrated transponder (PIT) tag (Digital Angel Corporation, Minnesota, USA) was injected into the left pelvic fin musculature to identify individual animals.

To obtain a qualitative estimate (i.e. without utilizing a population size model) of the minimum size of the stingray population at SCS and assess site fidelity of animals, we sampled SCS monthly and control sites weekly from February to May 2002 and April to August 2003, and additionally once each in September 2002 and January 2003 (i.e. a total of 11 census surveys were conducted at SCS between February 2002 – August 2003). At each SCS sampling event, stingrays were scanned for PIT tags, with previously uncaptured animals (judged by the absence of a PIT tag reading and left pelvic fin remnant scar – see below) receiving PIT tags. Stingray capture effort at each of the 11 sampling events was conducted over 3–4 days until no new (i.e., untagged) stingrays were encountered. When tagged animals were recaptured their location was recorded and the straight line distance traveled from tagging site determined. Tissue samples for a separate genetics investigation were taken from the left pelvic fin of every animal captured for the first time, and the remnant scars used both as a visual indicator of a previously captured animal and to determine PIT tag retention rates.

### Manual Telemetry

Seven fed stingrays (five mature females, two mature males), and six wild stingrays (six mature females) (Table 1) were tracked using manual acoustic telemetry methods. Maturity was assessed directly for males based on calcification of claspers, and for females based on disc width corresponding to size at maturity criteria from [28]. Mature females were chosen for the majority of manual tracks because they were the dominant demographic (>80% of individuals captured) at SCS. For manual tracking, external transmitters (V16-4H-01, Vemco, Nova Scotia, Canada, 16 mm diameter x 65 mm, 10 g in water, frequencies 51–81 kHz, lifespan 218 d) were attached to the right pelvic fin using a Peterson disk tag following the method of [29]. Handling time (capture to release) for each animal did not exceed seven minutes. Tracking was conducted from a 7 m boat equipped with a hull-mounted directional hydrophone (Vemco model VH10) and portable receiver (Vemco model VR60-01-02-07-08) crewed by a minimum of two people: a tracker/driver and an assistant responsible for anchoring and navigation [30]. Stingrays were manually tracked from 11to 72 h (Table 1) and their position recorded every ten minutes using a handheld GPS, with a total of 2,542 geographic positions recorded. Individual stingrays manually tracked for up to 72 h were followed for 3 non-contiguous 24-hr periods. All stingrays with external transmitters attached were released in good condition, judged by observations that the fed animals immediately returned to tourists to receive food handouts, and the wild stingrays swam off robustly after release.

**Table 1:** Fed and wild southern stingray individuals tracked by manual telemetry at Grand Cayman.

Stingray No.	Sex	Disc width (cm)	Site	Track start date	Track duration (hrs)
*Fed Stingrays*					
1	F	106.0	Stingray City Sandbar	26-Feb-02	24
2	F	102.0	Stingray City Sandbar	12-Mar-02	24
3	F	104.0	Stingray City Sandbar	19-Apr-03	72
4	F	124.5	Stingray City Sandbar	3-May-03	24
5	F	107.5	Stingray City Sandbar	28-May-03	72
6	M	58.0	Stingray City Sandbar	3-Mar-02	24
7	M	70.5	Stingray City Sandbar	14-Mar-02	24
*Wild Stingrays*					
8	F	99.0	South Sound	20-Mar-02	24
9	F	79.5	South Sound	2-May-02	24
10	F	89.0	South Sound	19-Jul-03	48
11	F	106.0	South Sound	30-Jul-03	48
12	F	90.0	South Sound	27-Aug-03	24
13	F	81.0	Rum Point	27-May-02	11

### Automated Telemetry

**Figure 2:**
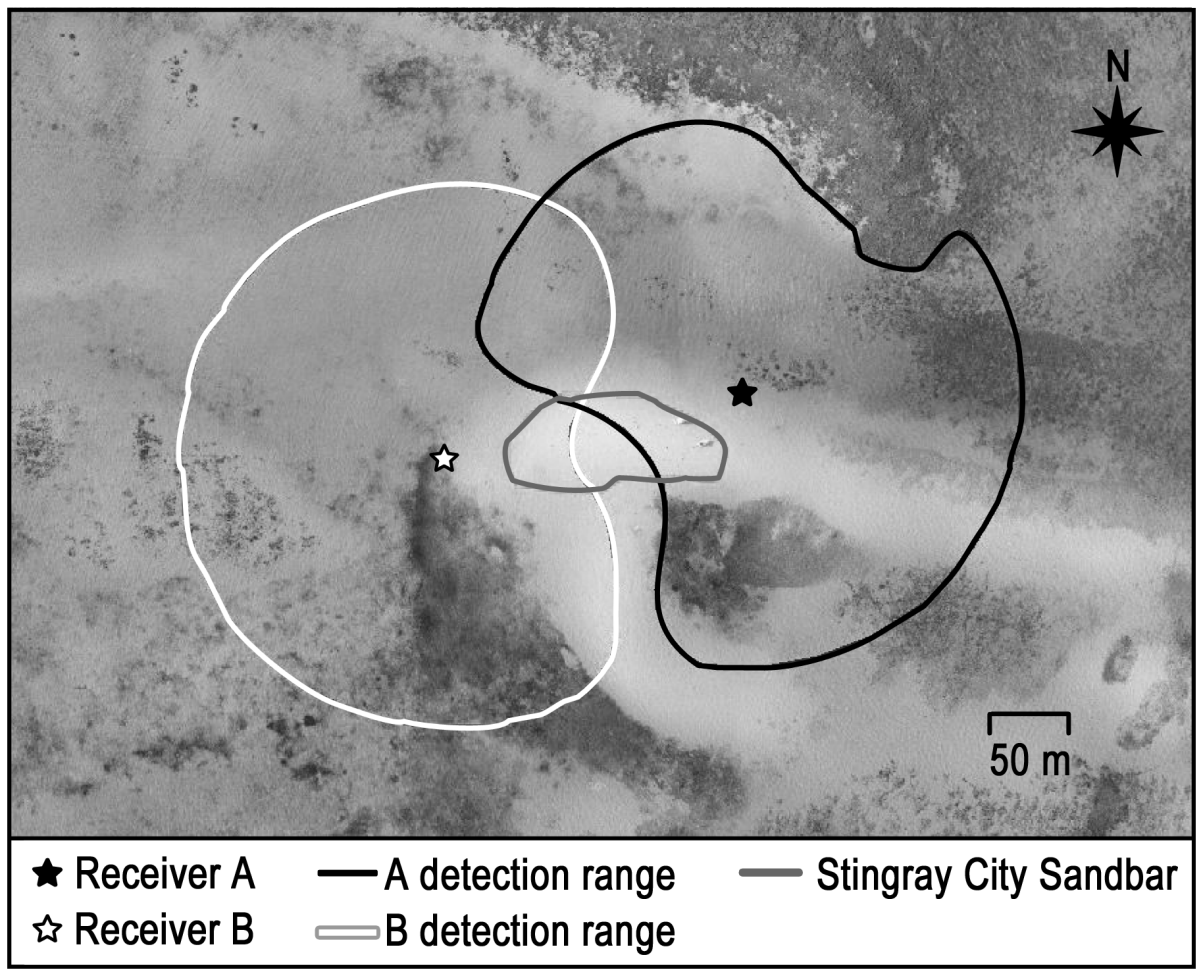
Locations and approximate detection ranges of two bottom-fixed, acoustic receivers. The receivers were used for longer-term, automated tracking of five mature female fed southern stingrays at Stingray City Sandbar (SCS), Grand Cayman.

We assessed longer-term (up to 389 days) site fidelity and movement patterns of the fed, mature females at SCS through use of automated acoustic receivers (Vemco model VR2-69.0 KHz-1.03-2-1431-C-211). Individually coded transmitters (V16-4H-01-R04K, Vemco 16 mm × 65 mm, 10 g in water, frequency 69 kHz, random pulse rates, lifespan 570 d) were coated with a thin layer of wax (50% beeswax, 50% paraffin wax) and surgically implanted in five mature females (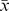 = 102.2±8.0 cm DW) (Table 2). Transmitters were inserted in the body cavity through a 20 mm incision in the ventral surface and the incision closed with four stitches with non-absorbable silk sutures. Two acoustic receivers encased in PVC housing for protection were anchored to the bottom 180 m apart on opposite sides of SCS at a depth of 3.5 m (Fig. 2). Range tests determined that the effective listening radius of the receivers was approximately 190 m, covering approximately 70% of the SCS supplemental feeding area. Receiver A was deployed for 389 days and receiver B for 202 days due to damage caused by a boat.

**Table 2:** Sex, size and detection duration of fed stingrays implanted with acoustic transmitters and monitored using two automated receivers deployed at Stingray City Sandbar (SCS), Grand Cayman.

Stingray No.	Sex	Disc width (cm)	Site	Monitoring start date	Total detection days
1	F	107	Stingray City Sandbar	08-Jul-03	389
2	F	98	Stingray City Sandbar	08-Jul-03	389
3	F	97	Stingray City Sandbar	11-Jul-03	386
4	F	114	Stingray City Sandbar	11-Jul-03	386
5	F	95	Stingray City Sandbar	14-Aug-03	353

### Data Analysis

Stingray tracks obtained by manual telemetry were overlaid on a photo-mosaic image of Grand Cayman Island using Arc View 3.2 GIS software. Stingray “core areas” (i.e. the area most frequently used by an animal) were calculated using 50% kernel utilization distribution, and “activity spaces” (i.e. the area that an animal traverses in the scope of normal activities) were calculated using a 95% kernel utilization distribution with the Animal Movement Analyst Extension (AMAE) program for Arc View GIS. The percent daily overlap in daytime core activity areas for fed vs. wild female tracked stingrays was calculated by determining overlap between the combined 50% kernel utilization distribution for each animal. Stingray rates of movement (ROM) were calculated by dividing the distance between successive position fixes by the sampling time interval using AMAE. Activity space and ROM data for each animal were divided into daytime and nighttime periods based on local sunrise and sunset, and the data subsequently pooled into “fed” and “wild” groups for comparison. Pooled day, night and total 24 hr activity spaces and ROM were compared within and between each group using a Mann-Whitney U-test. Pooled activity spaces and ROM were also compared over periods of high, low, incoming and outgoing tides using a Kruskal-Wallis test. High and low tidal phases were defined as the periods from one hour before to one hour after maximum and minimum water level.

Automated telemetry data collected with the VR2 receivers were examined using VR2PC software (Vemco, Version 1.12). To assess the degree of site attachment for each animal tracked by this method, detections of individual transmitters on the two acoustic receivers were sorted into hourly bins, and the presence of rays at SCS expressed as a percent of days an animal was present over the entire monitoring period. To assess if there was an overall temporal periodicity in the presence of the five female stingrays at the SCS over the receiver deployment periods, we conducted a time series analysis using a Fast Fourier Transformation (FFT) with Hamming window smoothing in SigmaPlot 11.0. The FFT decomposes time-series data into component sinusoidal waves of different frequencies, with the size of the spectral peaks in the resulting periodogram indicating the relative strength of the periodic components [31]. The FFT was conducted for the five animals for each receiver and both receivers combined.

## Results

Over the course of the February 2002–August 2003 study period, we captured 164 unique stingrays at SCS and 55 unique stingrays at the control sites (total of 219 unique stingrays). Ninety four percent of fed stingrays were recaptured at least once, 87% were recaptured at least twice and some animals were recaptured up to 11 times over the 19 months at SCS, totaling 986 individual recaptures at this site. Based on presence of scars from prior genetic tissue sample removal, PIT tag retention in recaptured animals at SCS was 100% during this period. With the exception of one animal, all recaptures at SCS were of stingrays originally tagged at this site. In contrast, only 22% of the wild stingrays were recaptured, all within 1 km of their original tagging sites.

**Figure 3:**
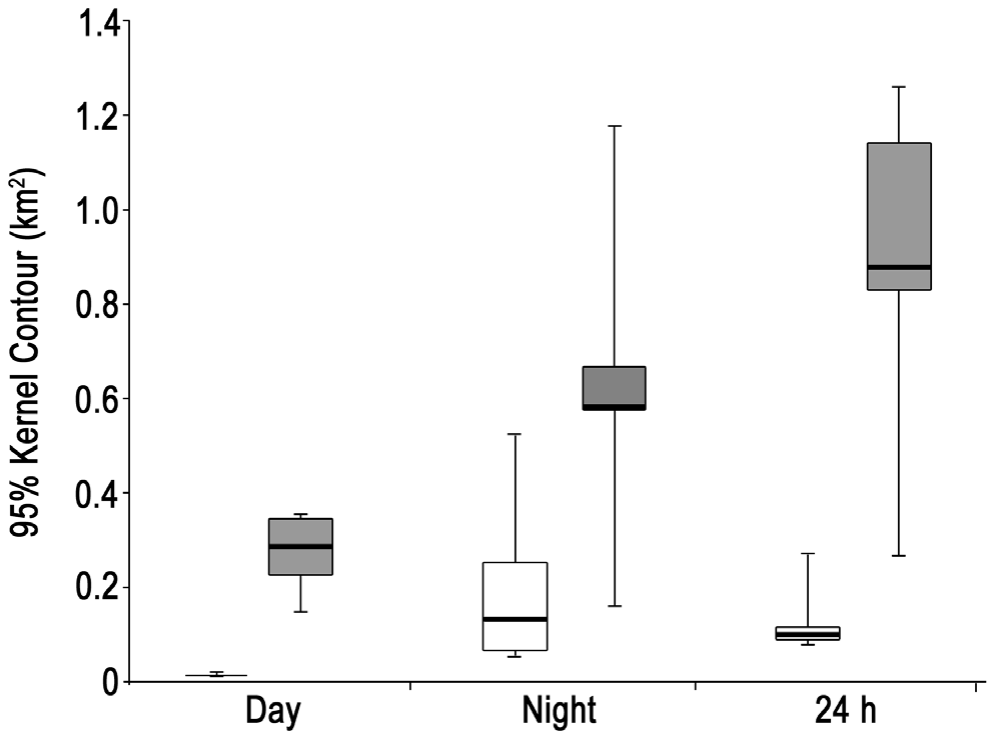
Comparative activity space sizes of fed and wild female stingrays. Activity space sizes, based on 95% kernel contours, of fed (open bars, n = 5) and wild (filled bars, n = 5) southern stingrays tracked manually at Grand Cayman during day, night and 24 hour periods. The thick horizontal lines inside the boxes represent the medians, the box edges show the upper and lower quartiles, and the whiskers represent minimum and maximum values.

**Figure 4:**
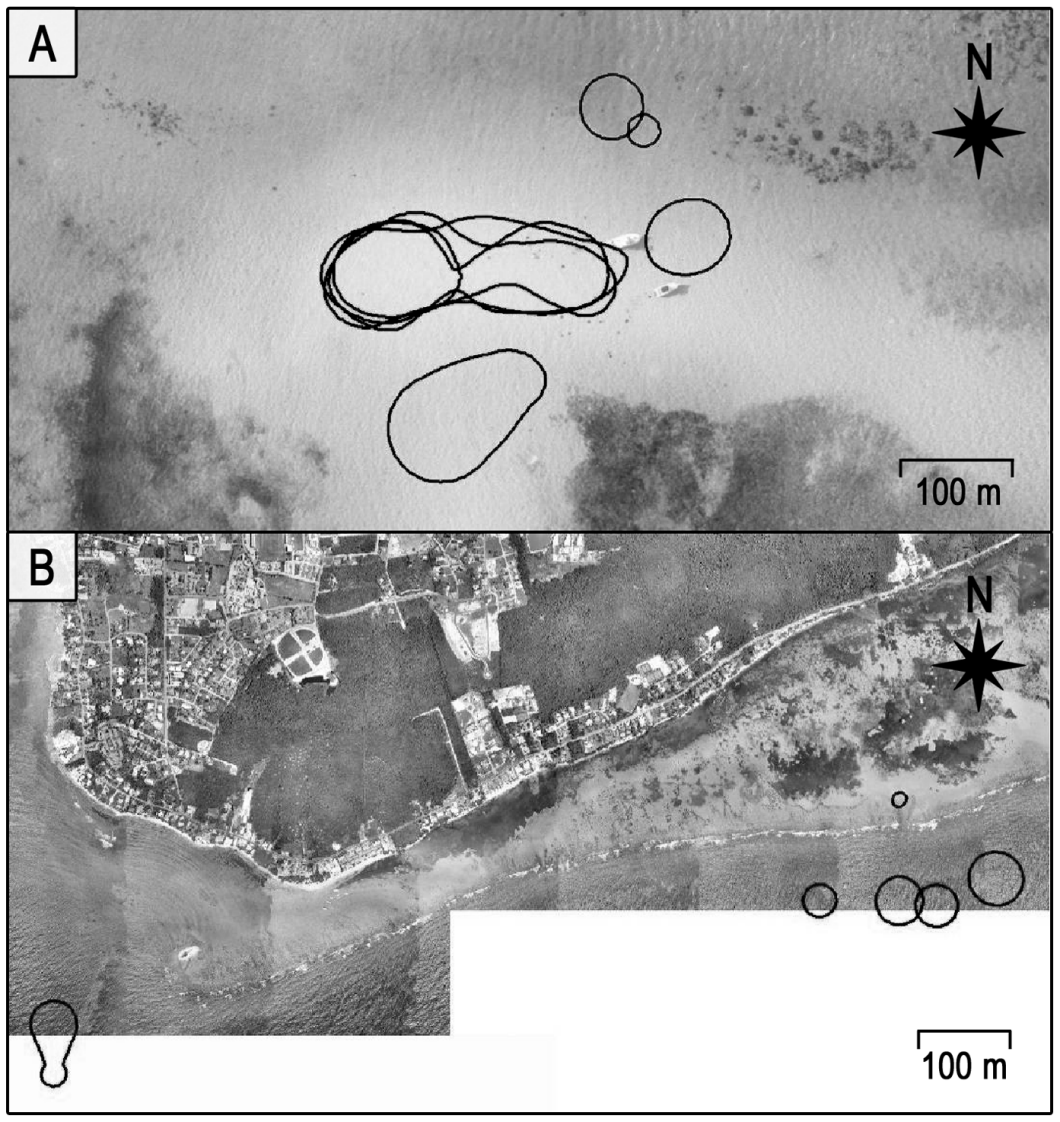
Comparative overlap in daytime core activity areas of fed and wild female stingrays. Core areas are based on 50% kernel contours of five stingrays manually tracked at each site. A) the Stingray City Sandbar (SCS) supplemental feeding site, and B) the South Sound control site.

Overall, there were several strong contrasts in movement and habitat use patterns between fed and wild stingrays: 1) Fed female stingrays had significantly smaller average daytime, nighttime and total (24 hr) activity spaces (total activity space = 0.13±0.08 km^2^) than wild female stingrays (total activity space = 0.88±0.17 km^2^) (Mann Whitney U test, P<0.01) (Fig. 3), 2) Fed females were much more active (i.e. constantly moving) during the day and much less active during the night than wild females, and 3) There was substantially greater overlap in daytime core space use by the fed compared to wild female stingrays (72% vs 3%, respectively; Fig. 4). On the other hand, there was no significant difference in ROM either within fed or wild stingrays or between these groups (Mann-Whitney U-test, P>0.05). Tidal phase had no influence on activity space or ROM for either group (Kruskal-Wallis test, P>0.05).

### Fed Stingray Movements – Manual Telemetry

All the tracked fed female stingrays remained active (i.e. displayed almost continuous movements without stationary periods) at SCS during daytime supplemental feeding periods. In addition to their telemetry tracked movements, the continuous activity of the tagged stingrays was also easily visually observable due to the shallow depth (0.5–1.5 m) and water clarity at the SCS. Less than one hour after cessation of daily supplemental feeding, which normally ended around 1700 h with the departure of ecotourism operators, all manually tracked female stingrays moved to the adjacent patch reef site ∼ 200 m north of SCS (Fig. 1); here they buried in the sand and remained stationary for several hours among aggregations of 20–30 individuals, with their heads oriented into the current. Between 1930 and 2130 h, the female stingrays moved from this aggregation area to individual “resting” locations within a 600 m radius of SCS where they sat stationary on the bottom with little to no further movement for several hours (stationary phase average ± sd = 5.8±1.9 hrs). Stingray individuals 3 and 5 (Table 1) were tracked for 72 hours and both exhibited strong fidelity to the same nighttime “resting” locations each night.

**Figure 5:**
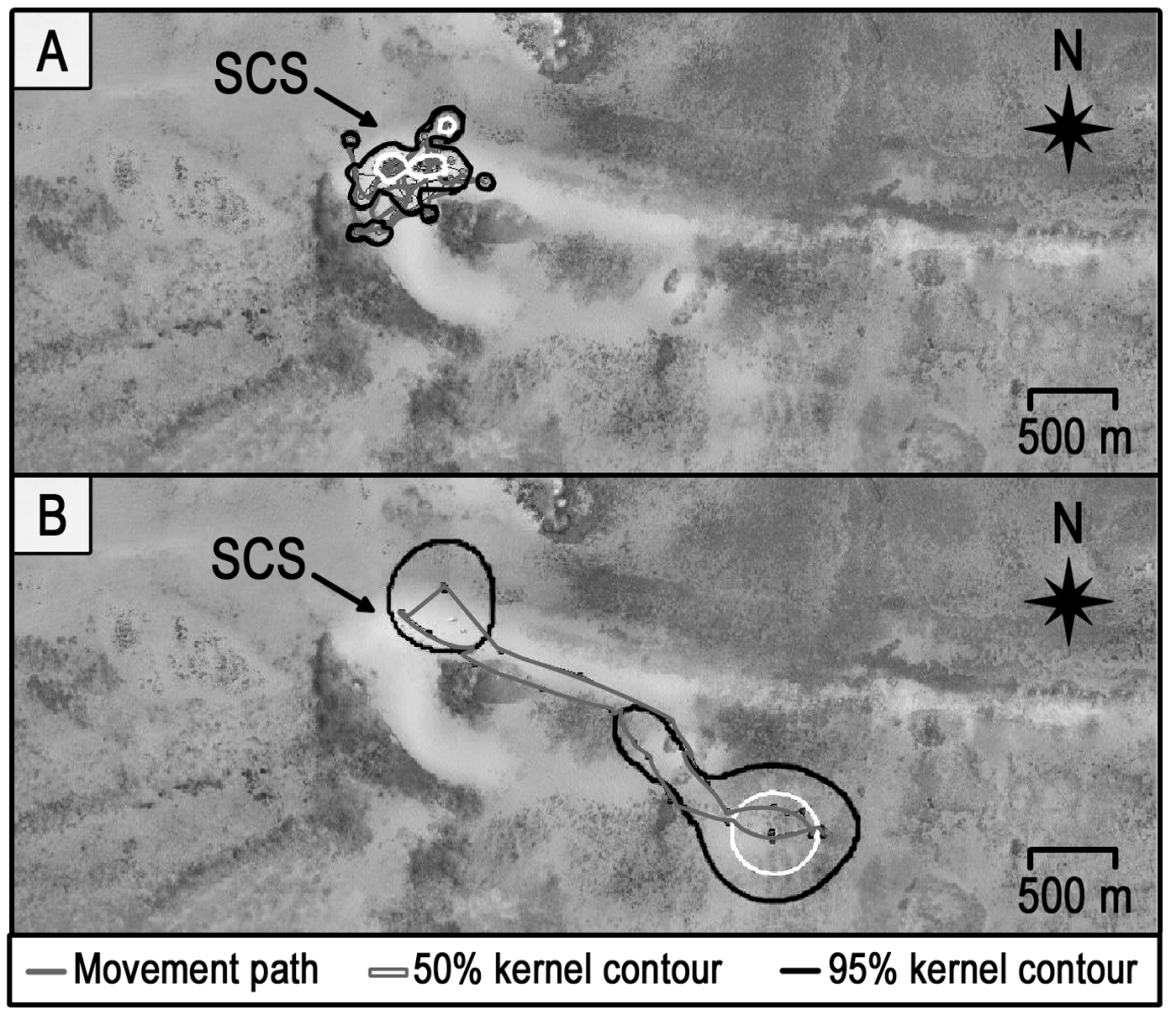
Comparative daytime vs. nighttime core areas (50% kernel contours) and activity spaces (95% kernel contours) of fed mature female stingray number 4 at Stingray City Sandbar (SCS). Panel A) daytime; panel B) nighttime. The movement behavior displayed by stingray number 4 is representative of the behavior of the four other fed females tracked at this site.

All tracked stingrays returned to SCS at least one hour prior to arrival of tourist boats and commencement of supplemental feeding the following day (around 0600 h). Fed female stingrays had significantly larger nighttime (0.21±0.19 km^2^) than daytime activity spaces (0.014±0.003 km^2^) (Mann-Whitney U-test, *P*<0.05) (Fig. 3), even though the stingrays were much more mobile during the day than night (Fig. 5).

The two fed, mature male stingrays (individuals 6 and 7; Table 1) tracked manually also remained active at SCS during daytime supplemental feeding activities. However, in contrast to the fed females, both males moved northeast 300 m to the shallow fringing reef crest following the cessation of supplemental feeding. Stingray individual 6 stopped at the reef and remained stationary for 2 h 35 m and stingray individual 7 circled the reef. Both individuals moved westward over the rubble flat zone, parallel to the fringing reef for approximately 2 km. Between 0015 h and 0025 h both males stopped moving along the fringing reef and remained stationary either within or adjacent to the reef for approximately 5 h 30 min, before traveling back to SCS shortly prior to the commencement of supplemental feeding the following day. As a result of their more extensive nighttime movements, the two fed males had a much larger average nighttime activity space (1.23±0.49 km^2^) than the five fed females (0.21±0.19 km^2^). However, the average daytime activity space of the tracked males (0.033±0.006 km^2^) was only slightly larger than that of the tracked females (0.014±0.003 km^2^). Male and female activity spaces were not statistically compared due to the small sample size for males (n = 2).

### Fed Stingray Movements - Automated Telemetry

All five stingrays with surgically implanted transmitters were released in good condition as judged by their return to tourists to receive food handouts immediately upon release. Incisions made for transmitter implantation appeared healed within 20 days of surgery. All five stingrays were detected on one or both receivers for the 353 to 389 days they were carrying transmitters, indicating that transmitter retention and stingray survival were 100%. All the animals were recorded on one or both receivers for at least a portion of every daytime period of the study, demonstrating that fed stingrays exhibit 100% high site fidelity to the small area at SCS. The FFT analyses with both the individual and combined receiver data demonstrated a clear dominant peak corresponding to a 24 hr detection periodicity for all five stingrays at SCS (not shown) throughout the receiver deployment period (202–389 days). Much smaller, secondary peaks corresponding to a 12 hr periodicity were also evident for some animals; however, their low strengths relative to the 24 hr peaks were suggestive of a harmonic artifact rather than a signal of true temporal periodicity.

**Figure 6:**
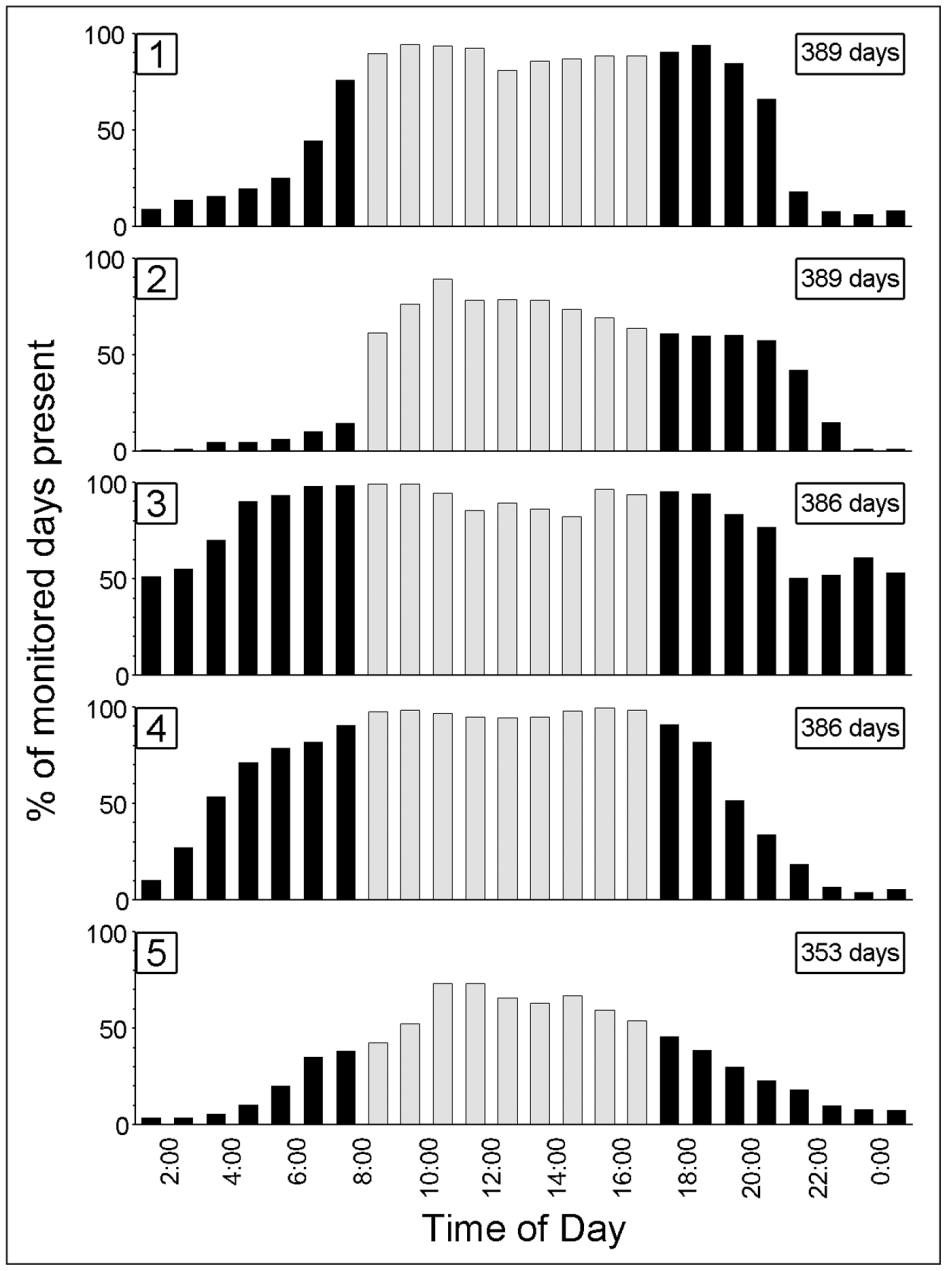
Diel detections of five mature female stingrays on bottom-fixed, automated acoustic receivers at Stingray City Sandbar (SCS). Percent values (y-axis) represent the number of days an individual stingray was detected on at least one receiver within the hourly time interval shown (x-axis). The values are expressed as a percent of the total monitored days (i.e., one or both receivers were present at SCS). Gray bars indicate typical times of ecotourism provided supplemental feeding.

Data from both receivers also showed a diel detection periodicity that was positively associated with daytime supplemental feeding activities (i.e. stingrays were present at SCS more frequently during supplemental feeding hours) (Fig. 6). The automated telemetry findings confirm the manual telemetry results, showing repeated and predictable use of SCS by stingrays during feeding events, suggesting that these patterns are typical diel movements and exhibited for prolonged periods of time.

### Wild Stingray Movements - Manual Telemetry

**Figure 7:**
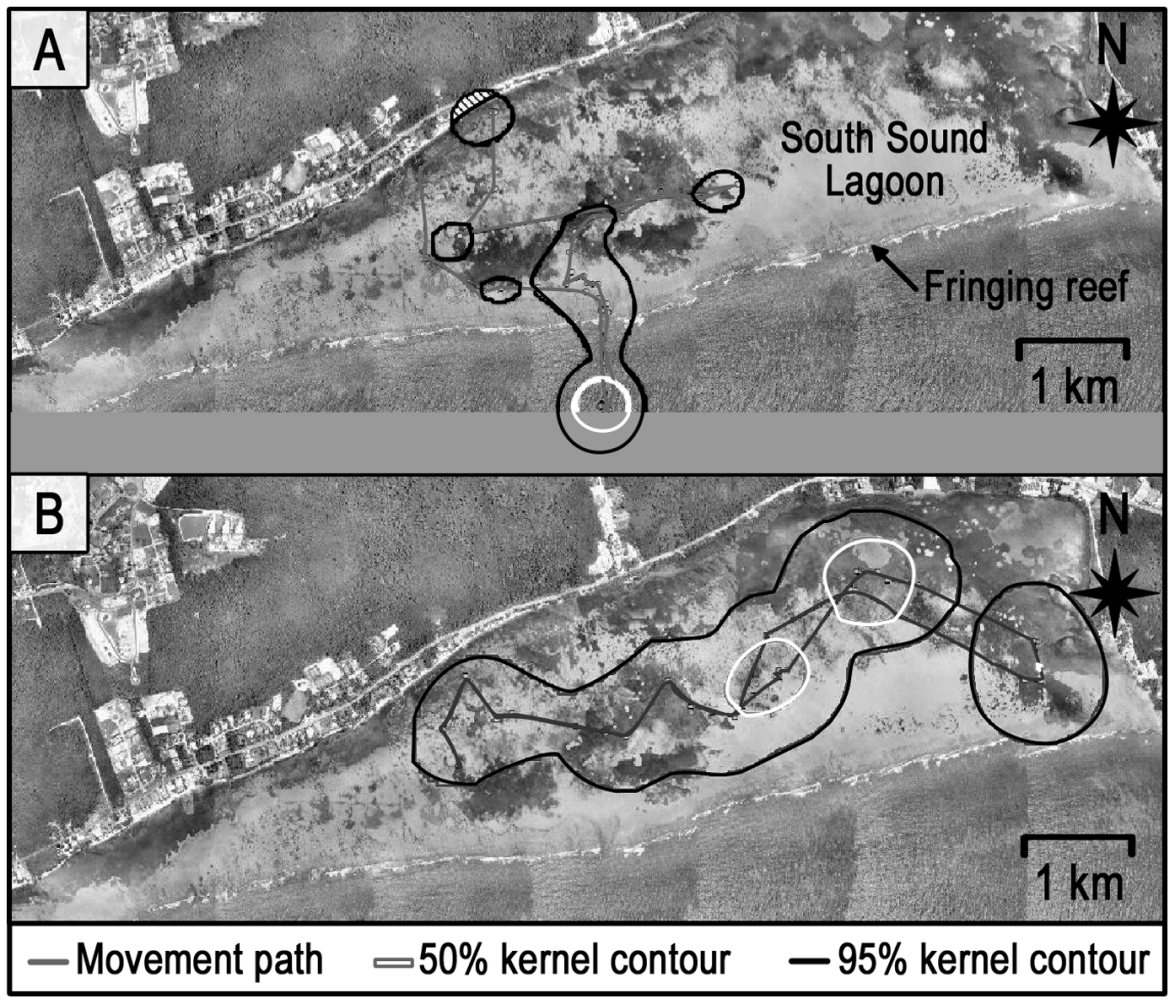
Comparative daytime vs. nighttime core areas (50% kernel contours) and activity spaces (95% kernel contours) of wild mature female stingray number 10 at the South Sound control site. Panel A) daytime; panel B) nighttime. The movement behavior displayed stingray number 10 is representative of the behavior of the four other wild females tracked at this site.

Wild female stingrays (n = 5) manually tracked in the South Sound lagoon all behaved similarly, with limited movements during the day and much more extensive movements at night. All the females exited the South Sound between 1000 and 1100 h where they spent a minimum of 4 h 15 min lying stationary on the bottom in >15 m depth (Fig. 7). Stingray individuals 10 and 11 (Table 1), each tracked for 48 hrs (two non-contiguous 24 h periods), showed fidelity to specific daytime locations outside the fringing reef on both days. No foraging by the tracked stingrays was observed during most (see below) of the day, when they were mostly stationary with the exception of traveling to and from the lagoon. However, the tracked and several non-tracked wild stingrays were observed foraging during early morning (0500–0700 h) and nighttime periods inside the lagoon over sand flat and grass plain zones. All five tracked wild females moved back into the lagoon from outside the fringing reef between 1515 and 1730 h and foraged over relatively large areas at night; subsequently, they had significantly larger activity spaces at night (0.63±0.36 km^2^) than during the day (0.27±0.09 km^2^) (Mann-Whitney U-test, P<0.05) (Fig. 3).

The single wild female stingray (individual 13, Table 1) tracked for 11 h (starting at 1350) at a secondary control site (Rum Point; Fig. 1) provided limited additional information on movement patterns away from the SCS. The female remained stationary during the afternoon, but moved continuously throughout most of the night (tracks not shown). Although the track was of relatively short duration, the behavior of this female was consistent with the pattern of limited movements during the day and more activity during the night observed for wild female stingrays in the South Sound.

## Discussion

The SCS site in the Cayman Islands is a renowned stingray interaction venue hosting very large numbers of tourism visitors who provide almost daily feeding to the animals. We examined the effects of this long-term (since 1986) feeding interaction on the movement behavior and residency of the stingrays at this site, and provide an initial perspective on the demographics of this population of ecotourism-conditioned animals. Acoustic telemetry and PIT tagging results indicated that the fed stingrays at SCS represent a spatially distinct population from wild stingrays, and that little mixing of animals from the two groups occurs. Based on the high frequency of animal recaptures and infrequent occurrence of new, untagged animals in the 11 sequential surveys conducted over 19 months, we qualitatively estimate that a population of at least 164 *Dasyatis americana* utilized SCS during the study period. We note, however, that our sampling surveys provide a minimum population size estimate. A quantitative, population model-based estimate of the overall stingray population size at SCS is warranted based on longer term mark-recapture data (ongoing work by authors BW, GH and MS).

The majority (over 80%) of the stingrays utilizing SCS consisted of mature females, making this demographic the most affected by ecotourism interactions. The highly skewed gender ratio found at SCS contrasts with observations at the only other batoid ecotourism operation examined in a demographic context, where the researchers found a nearly equal proportion of males and females [11]. The reason for this strong gender bias at SCS is unclear, although competitive exclusion of the smaller males by the much larger females (a strong body-size gender dimorphism exists in *D. americana*) may play a role (also see below).

Among the key findings of our study are that the movement patterns, diel activity, and habitat use of the fed female stingrays differ considerably from wild female stingrays, and that these differences appear to be maintained over long periods of time. Thus, there is strong evidence that supplemental feeding of stingrays by humans at SCS has resulted in long-term, drastic alterations in the behavior of these large animals.

Patterns in the movements of fed and wild female stingrays became clear because of behavioral consistencies displayed on a daily basis by individual animals as well as stingrays from each group. *Dasyatis americana* is naturally a nocturnal species that forages at night [32]. The observation of nighttime activity space as significantly larger than daytime activity space in both groups is consistent with behavior typically displayed by nocturnal animals, including other congener stingray species [29], [33]. However, in contrast to the wild stingrays, the situation with fed stingrays at SCS was unusual because their total activity space was not indicative of the level of actual activity (motion) exhibited by each animal during diurnal and nocturnal periods. For example, although fed stingrays occupied very small activity spaces during the day at SCS, they demonstrated almost continuous motion within this small activity space, moving from one tourist boat to another in response to potential food provision. It is important to note that although nighttime activity space estimates for fed females were significantly larger than their daytime activity space, this was due to stingray movements away from SCS at night to adjacent resting areas, increasing the area traversed. We emphasize, however, that the fed stingrays were far less active in this larger space, remaining mostly stationary at night.

In contrast to the fed stingrays, the wild stingrays demonstrated an opposite diel pattern, foraging at night over large activity spaces but remaining mostly stationary during the day. The large difference in amount of space utilized by females of the two groups while searching for food is highlighted by the fact that the average activity space of the wild stingrays at night was ∼45X greater than the average activity space of the fed stingrays during the day (0.63±0.36 km^2^ vs. 0.014±0.003 km^2^, respectively).

The inversion of diel activity from nocturnal to diurnal foraging in the fed animals was strongly associated with the presence of food availability from tourists during the day. Such extreme diel inversions in activity, although seen in a few other taxa, are relatively rare presumably because physiological and ecological adaptations underlie the evolution of circadian activity rhythms [34]. The temporal inversion in activity demonstrated by the fed stingrays is further notable because it would likely have some ecological costs (e.g., competitor and predator interactions) [35]. The occurrence of some very young stingrays at SCS raises the question of whether this diel behavioral inversion has become a fixed activity rhythm inherited by the progeny of these stingrays, or is a socially learned behavior, a capability recently documented in an elasmobranch [36].

The small, daytime activity spaces with high degree of spatial overlap seen in the fed female *D. americana* at SCS is illustrative of the greatly increased density of individuals at the ecotourism feeding site. The consistency of this altered habitat utilization behavior in the fed females suggests that supplemental feeding at a spatially restricted site has allowed the stingrays to reduce and centralize their core areas of activity, while still maximizing food accrual. This finding is consistent with those from diverse taxa that females tend to restrict activity spaces to the smallest possible while meeting energetic requirements [37], [38], [39]. Although several studies investigating the supplemental feeding of terrestrial vertebrates have observed similar decreases in activity space of the fed animals [26], [27], [40], we are unaware of other studies that have quantitatively revealed a similar outcome in supplementally fed fishes.

Although the link between an unnatural food source and unnatural habitat utilization behavior of stingrays at SCS is evident, it is less clear why male and female fed stingrays might behave differently with regard to movement patterns and activity space sizes. Acknowledging that the small sample size of manually tracked fed male stingrays (n = 2) precludes robust inferences, the consistency in the behavior of both males suggests that marked differences between the behavior of male and female fed stingrays may occur. Although males and females were both constantly on the move and occupied small activity spaces during the day within SCS, the average nighttime activity space for males was much larger than that of females due to their much farther roving at night along the fringing reef. A plausible hypothesis for this behavioral difference is that the much larger females outcompete the males for food at SCS, and males may therefore be required to cover larger areas to supplement their diet by foraging at night. Competition for food and aggressive interactions among the rays at SCS is suggested by the numerous bite marks on the trailing edges of the pectoral fins of both sexes of fed stingrays [20]. Competition between genders for ecotourism provided supplemental food was also observed at a stingray feeding site in Australia, where the larger *Dasyatis* female stingrays behaved aggressively towards smaller conspecific males [9].

The much higher spatial overlap in habitat use demonstrated by individual female stingrays at SCS compared to wild stingrays suggests that the influence of supplemental feeding may extend beyond the behavior of individuals, and has likely also altered the normal population dynamics of *Dasyatis americana* at this highly visited, ecotourism site. Overlap of core areas of activity among individual animals is an indication of the density and patterns of spatial distribution or dispersion within a community [41]. Individual core areas of social vertebrate species commonly overlap one other [42], [43], [44], whereas individual core areas of solitary species rarely overlap [29], [45], [46], [47]. Supplemental feeding at SCS strikingly alters dispersion patterns of the stingrays from a normally solitary lifestyle [20] (this study) to that of very high spatial habitat overlap among a large number of individuals. This behavioral and habitat utilization shift has disrupted the normal spatial distribution and increased the local density of stingrays at SCS to unnaturally high levels, which may have fitness costs (see below). Similar increases in density of individuals due to the introduction of supplemental food have been recorded in a range of terrestrial mammals including coyotes [48], hares [49], primates [50], squirrels [51] and voles [52], [53].

The increased density of stingrays at SCS appears to have led to a much higher frequency of interactions and physical contact between conspecifics, and has been shown to result in increased disease transmission, parasite loading, altered blood chemistry, injuries and overall poorer body condition [20], [24], [25] (and M. Corcoran, personal observations). Furthermore, we speculate that under such long-term, highly crowded conditions there is the potential for alteration of the stingray mating system and reproductive patterns, as well as increased inbreeding. Although mature animals of both sexes are normally solitary, the high density of animals at SCS may result in a much higher frequency of encounters between the sexes in a small area; indeed a mature male might encounter over 100 mature females in a single day at SCS. Supporting the idea of a potentially altered mating system are the observations that stingray mating is frequently seen at SCS and gravid females appear to be present throughout the year [54] (and authors personal observations).

Other potential outcomes of the artificially induced, dense stingray aggregations include ecosystem level impacts. We did not study these ecosystem outcomes, but offer the following reasonable hypotheses for future testing based on our field observations. We have observed stingrays foraging during the daytime along the periphery of SCS when tourist traffic is absent or limited (e.g., bad weather days or in between cruise ship visits) resulting in supplemental food being unavailable or minimal for that day. Given the demonstrated influence of bottom-feeding batoids in structuring benthic communities [55], [56], [57], it is likely that even limited predation by over 160 stingrays clustered into a small area has the potential to abnormally modify the structure of the local benthic community.

The aggregation of stingrays may also unnaturally influence community structure not only by their role as predators, but also as prey. Large great hammerhead sharks (*Sphyrna mokarran)*, known predators of *D. americana* and other rays [58], are frequently observed in the vicinity of SCS (M. Corcoran, personal observations) presumably being attracted there in greater than normal numbers by “easy” prey (i.e., stingray) availability. Consistent with this supposition is that Semeniuk and Rothley [20] observed more than twice the number of obvious predator-inflicted wounds on stingrays at SCS compared to wild stingrays. It is not unreasonable, therefore, to hypothesize that human provision of food to the stingrays may have cascading effects on the population dynamics of both stingray predators and prey. Furthermore, the presence of dense stingray aggregations, thousands of tourists and a large quantity of food supplied by humans packed into a small area also represents a higher than normal, continuous supply of metabolic and nitrogenous wastes that cycle through and can influence the structure of benthic marine communities [59], [60]. Hence, the stingray feeding operation at SCS has the potential to alter functioning of the entire local marine ecosystem.

In summary, our study provides a quantitative assessment of the effects of intense, ecotourism sourced, supplemental feeding on *Dasyatis americana* at Grand Cayman, showing that it results in long-term alterations in individual stingray movement, habitat-use patterns including diel inversion of activity, and dynamics of their population. Our study also raises the reasonable possibility that the non-natural aggregations of the stingrays caused by supplemental feeding may have cascading effects on the surrounding marine ecosystem, impacting both stingray predator and prey dynamics. Our findings coupled with those of Semeniuk and colleagues [20], [24], [25] on the physiological and potential fitness costs incurred by fed southern stingrays at SCS leave little doubt about the major effects that the tourism sourced feeding operations are having on the biology of the target species. Because feeding of marine wildlife on a regular and sustained basis for tourism is widespread and continuing to expand, understanding the impacts of these activities on the target marine organisms and associated ecosystems will be useful to help managers plan mitigating measures where these activities exist, and exercise precautionary policies where new feeding sites are proposed.
